# Supplementary material for: Computational‐Design Enabled Wearable and Tunable Metamaterials via Freeform Auxetics for Magnetic Resonance Imaging
Source: Adv Sci (Weinh). 2024 Apr 24;11(26):2400261. doi: 10.1002/advs.202400261 (PMC11234395; doi:10.1002/advs.202400261)
Supplement: Supplementary file 1 — Supporting Information [file ADVS-11-2400261-s003.pdf]

ADVANCED SCIENCE
Open Access

Supporting Information

for *Adv. Sci.*, DOI 10.1002/advs.202400261

Computational-Design Enabled Wearable and Tunable Metamaterials via Freeform Auxetics for Magnetic Resonance Imaging

Ke Wu, Xia Zhu, Thomas G. Bifano, Stephan W. Anderson and Xin Zhang**

Supporting Information

Computational-design Enabled Wearable and Tunable Metamaterials via Freeform Auxetics for Magnetic Resonance Imaging

*Ke Wu, Xia Zhu, Thomas G. Bifano, Stephan W. Anderson *, and Xin Zhang **

This file includes:

Supplementary Text

Figures: S1 to S10

Table: S1

Other Supplementary Materials for this manuscript include the following:

Movie S1: A video showing the deploying process between contraction and expansion states of the auxetic configurated in kneecap shape.

Movie S2: A video showing the deploying process between contraction and expansion states of the auxetic configurated in ankle cap shape.

Table of Contents

Supplementary text 3

Section S1. Optimization procedure based on triangle CP mesh for a planar shape with freeform boundary. 3

Section S2. Optimization procedure based on quadrilateral CP mesh for a freeform surface approximating ankle cap. 4

Section S3. The optimization of the resonators for constructing metamaterials 5

Section S4. Mathematical modeling of a pair of magnetic helical coil resonators. 6

Section S5. Theoretically evaluating SNR performance 9

Supplementary Table 11

Table S1. Parameters employed in derivations for inductance and capacitance. 11

Supplementary Figures 12

Figure S1. Process flow for developing the tunable and wearable metamaterials for MRI application. 12

Figure S2. Function blocks diagram based on triangle CP mesh in Grasshopper. 13

Figure S3.. Optimization process based on the triangle CP mesh for a plane with freeform boundary. 14

Figure S4. Function blocks diagram based on quadrilateral CP mesh in Grasshopper. 15

Figure S5. Optimization process based on the quadrilateral CP mesh for a freeform surface approximating the ankle cap configuration. 16

Figure S6. Optimization for HCRs. 17

Figure S7. Geometric parameters employed in mathematical derivation for a pair of helical coils resonators. 18

Figure S8. Experimental setup for the EM characterizations of knee metamaterial (a) and ankle metamaterial (b). 19

Figure S9. Two-image method for SNR evaluation. 20

Figure S10. CST simulation model for knee metamaterial and ankle metamaterial. 21

Reference 22

Supplementary text

Section S1. Optimization procedure based on triangle CP mesh for a planar shape with freeform boundary.

The realization of the circle packing (CP) is a complex optimization problem requiring numerous iterations, especially for complex freeform surfaces. In this work, all optimization processes for obtaining CP patterns were conducted within Grasshopper, a visual programming interface integrated into the 3D modeling program Rhinoceros. Grasshopper facilitates parametric design by offering a wide array of functional integrations within an interactive and intuitive environment. Leveraging the features of Grasshopper, we were able to generate CP patterns for complex surfaces by addressing highly intricate problems in a hands-on manner. Several illustrative examples, showcased in Figure 1 in the main text, validate the effectiveness of this computational method. Our approach commenced with a planar surface with freeform boundary. The CP pattern was derived through a triangular CP mesh. Within Grasshopper, optimization programs were created by intuitively dragging components onto a canvas. The outputs of these components were then interconnected with the inputs of subsequent components, as illustrated in **Figure S1**. The optimization steps are outlined in **Figure S2**, and the detailed implementation process of circle packing is described below:

a). The general optimization process commences with an arbitrary planar surface, as depicted in Figure S2a.

b). The initial step involves generating a triangular mesh that approximates the boundary of the given planar surface, as illustrated in Figure S2b. The density of this mesh can be adjusted using input parameters such as the mesh edge length. Consequently, the mesh length serves as a controlling variable determining both the subsequent packing density and the number of unit cells in the final designed metamaterial. Prior to optimization, the inner circles of the triangles within the mesh were extracted. While all triangles are tangent to the mesh edges, the tangent points on the same mesh edge do not necessarily intersect at the same point, as depicted in Figure S2c. From these inner circles, outer circles (shown in green in Figure S2d) were generated at the tangent points between the inner circles and mesh edges. However, these outer circles are not initially tangent to each other. To ensure that the outer circles are tangent to one another, an optimization process is necessary, relying on the geometrical properties of the triangular CP mesh.

c). Subsequently, the surface boundary condition, along with the vertices and edges of the initial triangle mesh, were extracted as input parameters. Utilizing these extracted parameters, Kangaroo, a live physics engine designed for interactive simulation, form-finding,

optimization, and constraint solving within Grasshopper, was employed. Kangaroo optimized the triangle mesh based on the formulated constraints of the triangle CP mesh. Following the optimization process, an updated triangle mesh (indicated by the green edges in Figure S2e) was obtained from the initial triangle mesh (indicated by the red edges in Figure S2e).

d). Using the optimized triangular CP mesh, the TangentCircles component in Grasshopper was utilized to generate the inner circles of the triangles, as illustrated in Figure S2f. These inner circles confirm that all circles are tangent to the mesh edges, and the tangent points on the same edge intersect at the same point. This arrangement ensures that the outer circles, packed around the vertices, are tangent to each other.

e) Figure S2g illustrates the desired CP pattern formed by the outer circles for the given planar surface

f). Finally, the coordinates of the packing circles' center points, diameters, tangent points, and circle diameters (as demonstrated in Figure S2h) are extracted for the subsequent design of the deployable auxetic structures.

Section S2. Optimization procedure based on quadrilateral CP mesh for a freeform surface approximating ankle cap.

For surfaces exhibiting irregular shapes and significant curvature, a quadrilateral CP mesh was employed to achieve compact and highly tangential circle packing patterns. In the context of this study, our focus is on designing wearable metamaterials for MRI applications. As an example, a surface approximating an ankle cap was utilized to illustrate the optimization process based on the quadrilateral CP mesh. **Figure S3** presents the block diagram developed in Grasshopper for realizing the quadrilateral CP pattern. The implementation process is described in detail below:

a). Firstly, a scanned 3D model of the human body was imported into Rhinoceros. The specific area corresponding to the ankle cap was selected as the target surface for CP optimization, as depicted in **Figure S4**a. This selection allowed for the subsequent design of a metamaterial tailored for ankle imaging in MRI systems.

b). Figure S4b illustrates the selected surface approximating the ankle cap.

c). Given the selected surface, the initial step involved meshing the surface with a quadrilateral mesh, as demonstrated in Figure S4c.

d). Based on the formulated geometrical properties of the quadrilateral CP mesh described by Equation 2 in the main text, the total edge length on two opposite side should equals to each other for obtaining a quadrilateral CP mesh. As a result, to facilitate easier

convergence in the subsequent CP optimization, the surface was re-meshed with the requirement that the mesh edges' lengths roughly approximated each other. Simultaneously, during this step, the packing density was determined by inputting the mesh edge length. The initial optimization result is depicted in Figure S4d. To visually demonstrate the tangent condition of the incircles, the component "TangentCircles" was employed to plot the circles of the quadrilaterals in the mesh, as shown in Figure S4e. The cluttered arrangement of incircles indicates that the current mesh does not satisfy the geometrical properties of the quadrilateral CP mesh.

e). Next, the surface boundary condition, along with the vertices and mesh edges of the quadrilateral mesh (shown in Figure S4f), were extracted as input parameters for optimization.

f). Applying the formulated constraints, Kangaroo optimized the mesh, ensuring the tangent incircle property, surface fitting, and boundary constraints were satisfied. The optimized quadrilateral CP mesh, indicated by green edges in Figure S4g, was obtained from the initial mesh, shown by red edges.

g). The optimization result yielded the desired quadrilateral CP mesh, where each quadrilateral encapsulates an incircle, and the neighboring incircles are tangent to each other along the shared mesh edges, as illustrated in Figure S4h.

h). For complex surfaces, the optimization results often compromise the uniformity of incircles' diameters along the sides of the mesh. To mitigate this issue, one possible approach is to exclude the circles on the sides of the mesh, as demonstrated in Figure S4i. Lastly, the coordinates of the packing circles' center points, diameters, tangent points, and circle diameters were extracted for the subsequent design of deployable auxetics.

Section S3. The optimization of the resonators for constructing metamaterials

In the pursuit of optimal signal-to-noise ratio (SNR) enhancement in wearable metamaterials, the optimization of helical coil resonators (HCRs) plays a pivotal role in the metamaterial design. The magnetic metamaterial discussed in this study consists of an array of metallic HCRs. The coupling of these HCRs results in a synergistic effect, leading to collectively resonating modes. When excited by an external RF field, electric currents are induced along the metallic coils within the HCRs. At a specific resonance mode, these induced currents result in a significant enhancement of the RF field. Capitalizing on this property, the integration of metamaterials into MRI systems leads to a substantial improvement in the local RF MRI signal, ultimately resulting in significant gains in SNR. HCRs offer several advantages due to their unique configuration, as depicted in **Figure S5**a, including compact sizes, high Q

values, and ease of fabrication. A notable property of HCRs is their easily tunable resonant frequency through adjustments in their geometrical configurations. This design flexibility of resonance frequency is particularly crucial in MRI applications where precise frequency control is essential. The number of turns, the gap between neighboring turns, and the diameter of an HCR are variables that influence its resonance frequency. Figures S5b, S5c, and S5d depict contour lines representing points of equal resonance frequency for HCRs with diameters of 20, 30, and 40 mm, respectively. The resonance frequency of an HCR, indicated by the embedded number in contour lines, is a function of the number of turns and gaps. This relationship demonstrates that the desired resonance frequency can be achieved with various diameters and heights, allowing for precise tuning according to specific MRI requirements. However, HCRs with the same resonance frequency can exhibit different magnetic field enhancement performances due to their distinct Q values resulting from varied configurations. As the fundamental unit of metamaterials, the magnetic field enhancement capability of HCRs is a pivotal factor influencing the performance of metamaterials in MRI applications. Considering the MRI application scenarios, we conducted simulations of magnetic field strength at the center point of HCRs' top surfaces, which could be employed to indicate the HCR’s resonant oscillating strength. During these simulations, we varied the height of the HCRs (3, 5, 10, 15, 20, 30, and 50 mm), while keeping the HCRs' diameter constant at values of 20, 30, or 40 mm, respectively. The results, as depicted in Figure S5e, indicate that the HCR achieves optimal performance when its height is approximately 10 mm, when the HCR diameter is within 20~40 mm. Heights that are too high or too low result in a decrease in its Q value, weakening its field enhancement effect. Next, we compared the field enhancement performance of HCRs with different diameters. Based on the results in Figure S5e, we selected HCRs with geometrical properties of D20-H10, D30-H10, and D40-H10 (D and H indicate the diameter and height of the HCR, respectively) for the comparisons. The field enhancement ratios along the central axis of the HCRs were simulated and plotted in Figure S5f. As the diameter of the unit cells increased from 20 mm to 40 mm, the penetration depth improved at the expense of the maximum field strength at the coil surface. Investigating the tradeoff between penetration depth and SNR enhancement capabilities can serve as a guideline for choosing and optimizing the geometric configurations of HCRs when constructing metamaterials. This optimization can help maximize the SNR within the desired penetration depth for specific anatomical targets.

Section S4. Mathematical modeling of a pair of magnetic helical coil resonators.

In an effort to mathematically elucidate the coupling coefficients between a pair of two

inclined assembled helical coil resonators (HCRs) (as seen in **Figure S6**), the effective inductance and capacitance need to be derived. Initially, we employed Equation S1 below to define the effective self-inductance and mutual inductance of these two discrete unit cells.

$$L_{ij} = \frac{\mu_0}{4\pi|I_i I_j|} \iint dr_i dr_j \frac{J(r_i)J(r_j)}{|r_i - r_j|} \tag{S1}$$

In Equation S1, ri and rj represent integration elements along the path of the HCR, Ii and Ij are equivalent electric currents, and **J**(ri) and **J**(rj) denote the current densities (vectors) at ri and rj in the HCR. When i equals to j, Equation S1 allows for the calculation of the self-inductance of the HCRs. When i and j are different, Equation S1 provides the mutual inductance between these two HCRs. When HCR oscillates at its resonant state, the electric current's amplitude follows a sinusoidal profile along the helical wire, starting from 0 at the wire's two ends and reaching its maximum value at the middle of the wire. Utilizing this current profile, both the self-inductance and mutual inductance can be derived theoretically. Besides the effective inductance, the capacitance including both the mutual capacitance and the self-capacitance may be derived via the inverse of the coefficients of the potential matrix and be expressed as:

$$P_{ij} = \frac{1}{4\varepsilon_0\varepsilon_r|Q_i Q_j|} \iint dr_i dr_j \frac{\rho(r_i)\rho(r_j)}{|r_i - r_j|} \tag{S2}$$

$$\boldsymbol{C} = \begin{bmatrix} C_{11} & \cdots & C_{1m} \\ \vdots & \ddots & \vdots \\ C_{m1} & \cdots & C_{mm} \end{bmatrix} = \begin{bmatrix} P_{11} & \cdots & P_{1m} \\ \vdots & \ddots & \vdots \\ P_{m1} & \cdots & P_{mm} \end{bmatrix}^{-1} \tag{S3}$$

In Equation S2, Qi and Qj represent the equivalent charge amounts in the HCR, and ρ(ri) and ρ(rj) are the charge densities (scalars) at ri and rj. Due to the phase difference between the electric current and charge distribution being π/2, the charge density reaches its maximum at the ends of the wire (q0) and is zero at its mid-portion. The self-capacitance is determined by the diagonal elements of the capacitance matrix ***C***, while the remaining elements yield the corresponding mutual capacitance. Following the derivation of capacitance and inductance, the coupling factor *k* and the resonant angular frequency of a single HCR *ω0* can be readily calculated by solving the following equations:

$$\omega_0 = \frac{1}{\sqrt{L_s C_s}} \tag{S4}$$

$$k = k_L + k_C = \frac{L_m}{L_s} + \frac{C_m}{C_s} \tag{S5}$$

in which C_m and C_s represent the mutual capacitance and self-capacitance, while L_m and L_s represent the mutual inductance and self-inductance, respectively. Once the configuration of an HCR (diameter, number of turns, gap between neighboring turns, etc.) is fixed, the self-

inductance and self-capacitance become constant values. However, the mutual inductance and capacitance vary as a function of the separation distance between these two resonators, leading to the coupling coefficient being a variable value that changes with the separation distance. Figure 3a of the main text depicts the relationship between the total coupling coefficient of two resonators, as well as the contributions from capacitance coupling (k_C) and inductance coupling (k_L) as a function of separation distance. To mathematically investigate the reflection spectrum of a pair of HCRs, we employed coupled mode theory (CMT) to derive the frequency response under different coupling coefficients ranging from low to high. With the interunit cell coupling coefficient, the resonant modes of these two resonators can be derived using the following system of equations:

$$j\omega\begin{bmatrix}a_1\\a_2\end{bmatrix}=j\begin{bmatrix}\omega_1+j\left(\frac{1}{\tau_{e1}}+\frac{1}{\tau_{o1}}\right) & k\omega_1/2\\ k\omega_2/2 & \omega_2+j\left(\frac{1}{\tau_{e2}}+\frac{1}{\tau_{o2}}\right)\end{bmatrix}\begin{bmatrix}a_1\\a_2\end{bmatrix}+\begin{bmatrix}\sqrt{\frac{2}{\tau_{e1}}}\\ \sqrt{\frac{2}{\tau_{e2}}}\end{bmatrix}s_+ \qquad (S6)$$

In the Equations S6, the subscripts ‘1’ and ‘2’ indicate the two resonators. a_n (where n=1, 2) represents the mode amplitude of the resonator, ($1/\tau_{en}+1/\tau_{0n}$) denotes the decay rates of the oscillating strength of the resonators due to radiation and intrinsic losses, ω_n represents the resonance frequency, s_+ is a harmonic excitation signal function with frequency ω (i.e., $s_+=|s_+|e^{j\omega t}$), and $\sqrt{2/\tau_{en}}$ is the coefficient expressing the degree of coupling between the resonator and the excitation signal. Finally, given the mode amplitudes of these two resonators, the reflection spectrum of the array can be expressed by:

$$r=-1+\frac{\sqrt{\frac{2}{\tau_{e1}}}a_1+\sqrt{\frac{2}{\tau_{e2}}}a_2}{2|s_+|} \qquad (S7)$$

The theoretical reflection spectra with different coupling coefficients are presented in Figure 3b of the main text. All the parameters used in the derivation process are listed in Table S1. It's important to note that the discrepancy in radiation loss between τ_{e1} and τ_{e2} of the two coils is intentional and is meant to mimic real practical scenarios where the two HCRs have slightly different coupling coefficients with the excitation power. Without this discrepancy, the resonance mode where the two coils have opposite directions would not appear in the reflection spectrum. In reality, equality between τe1 and τe2 occurs only when the two resonators interact precisely the same way with the excitation power. By utilizing the parameters listed in Table S1, the theoretical results closely align with the measured results, indicating a strong agreement between theory and experimental observation.

Section S5. Theoretically evaluating SNR performance

To theoretically evaluate the SNR performance when metamaterial is applied to MRI, the underlying mechanism of SNR was explored by deriving the original governing equation for SNR in MRI:[S1,S2]

$$\mathrm{SNR} \propto \frac{\omega^2 B_c}{\sqrt{R_{BC} + R_{sample} + R_{mm}}} \tag{S8}$$

In Equation S8, Bc represents the magnetic field strength generated by the electric current in the receiving coil, and ω stands for the Larmor frequency. R_{BC}, R_{sample}, and R_{mm} in Equation S8 denote the series resistance arising from the receive coil, the sample, and the metamaterial, respectively. R_{BC} results from the conductive loss of the receive coil, while R_{sample} represents the power loss in the sample or patient due to the induced eddy currents. Since the metamaterial itself consumes energy and generates heat due to the induced current from the RF magnetic field, R_{mm} is employed to represent this power dissipation. These three elements constitute the principal noise sources in MRI. Obtaining an accurate estimation for the magnetic field strength and effective series resistance is generally complicated, especially when dealing with a sample having a complex geometry and an irregular distribution of the RF field due to the introduction of a metamaterial. In this study, a numerical simulation tool CST was employed to develop a simple and straightforward method for precisely evaluating the SNR in MRI. Since the Larmor frequency ω is directly proportional to the static field strength B0, it remains a constant value for the experimental setup reported herein. Consequently, the analytical SNR enhancement ratio can be calculated as the ratio of the simulated magnetic field strength divided by the square root of the total power dissipation in the birdcage coil (BC), phantom, and metamaterial, as expressed by:

$$\mathrm{SNR} \propto \frac{\omega^2 B_c}{\sqrt{P_{BC} + P_{sample} + P_{mm}}} \tag{S9}$$

All the parameters in Equation S9 can be readily extracted from the simulation results. To accurately evaluate the SNR through the numerical simulation method, a high-pass BC was initially constructed with the same geometry as the Philips 3T MRI scanner. This BC was designed to generate a homogeneous circularly polarized magnetic field, mimicking the B1 field in actual MRI environments. The 1% agarose gel phantoms were modeled using materials shaped like knee and ankle caps, with a relative permittivity of 78, electric conductivity of 0.12 S/m, and a material density of 1000 kg/m^3. The simulation models for the knee and ankle metamaterials are illustrated in **Figures S9**a and b, respectively. The analytical results, plotted in Figures 5d and i in the main text, were compared with the MRI experimental results,

demonstrating a high degree of agreement.

Supplementary Table

Table S1. Parameters employed in derivations for inductance and capacitance.

Parameter	Description	Value
R1/R2	Radius of helical coil	15 mm
gap	Gap between two coils	1.25 mm
n	Number of turns	7.25
θ	Inclined angle	30°
Dis	Separation distance	32~42 mm
$\tau01$	Intrinsic loss of coil_1	$1000/\omega_0$
$\tau e1$	Radiation loss of coil_1	$500/\omega_0$
$\tau02$	Intrinsic loss of coil_2	$1000/\omega_0$
$\tau e2$	Radiation loss of coil_2	$175/\omega_0$

Supplementary Figures

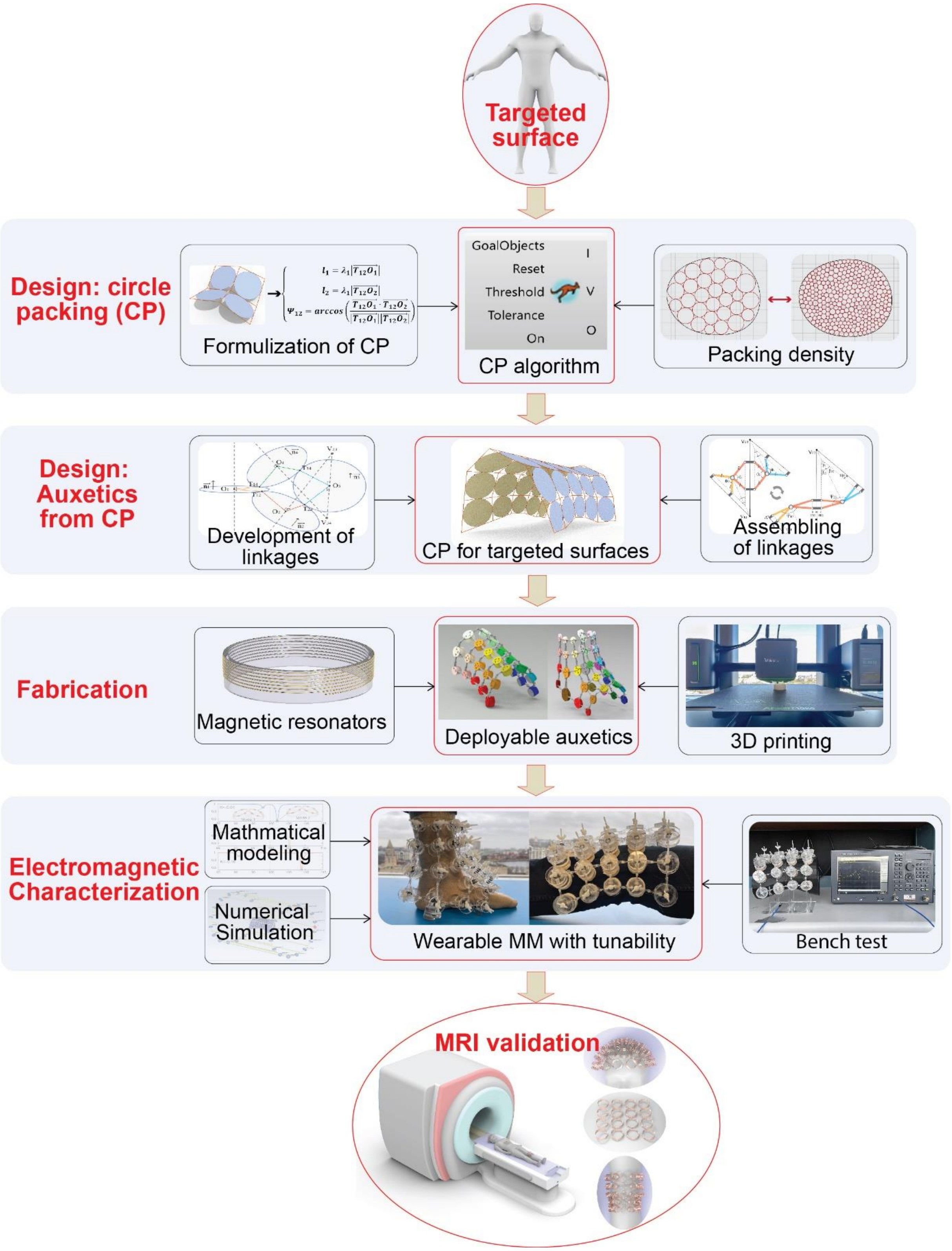

Figure S1. Process flow for developing the tunable and wearable metamaterials for MRI application.

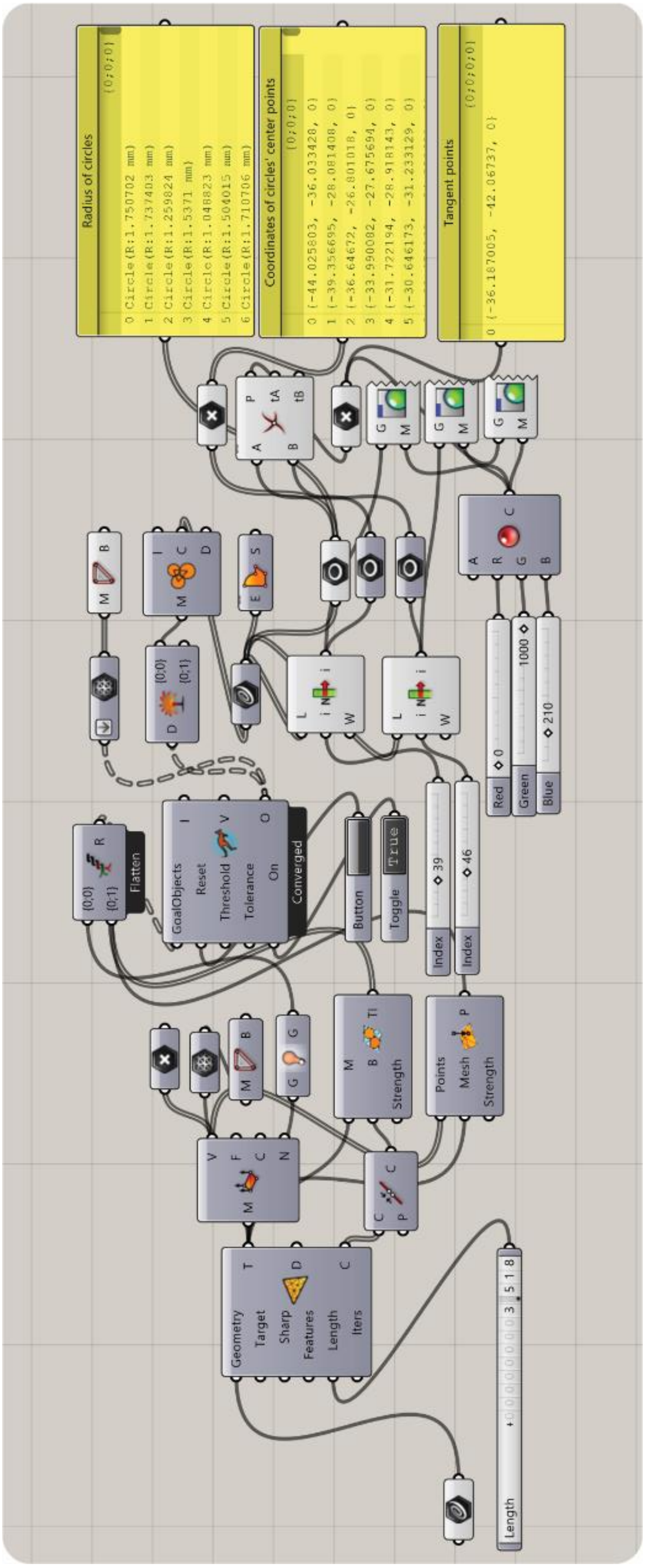

Figure S2. Function blocks diagram based on triangle CP mesh in Grasshopper.

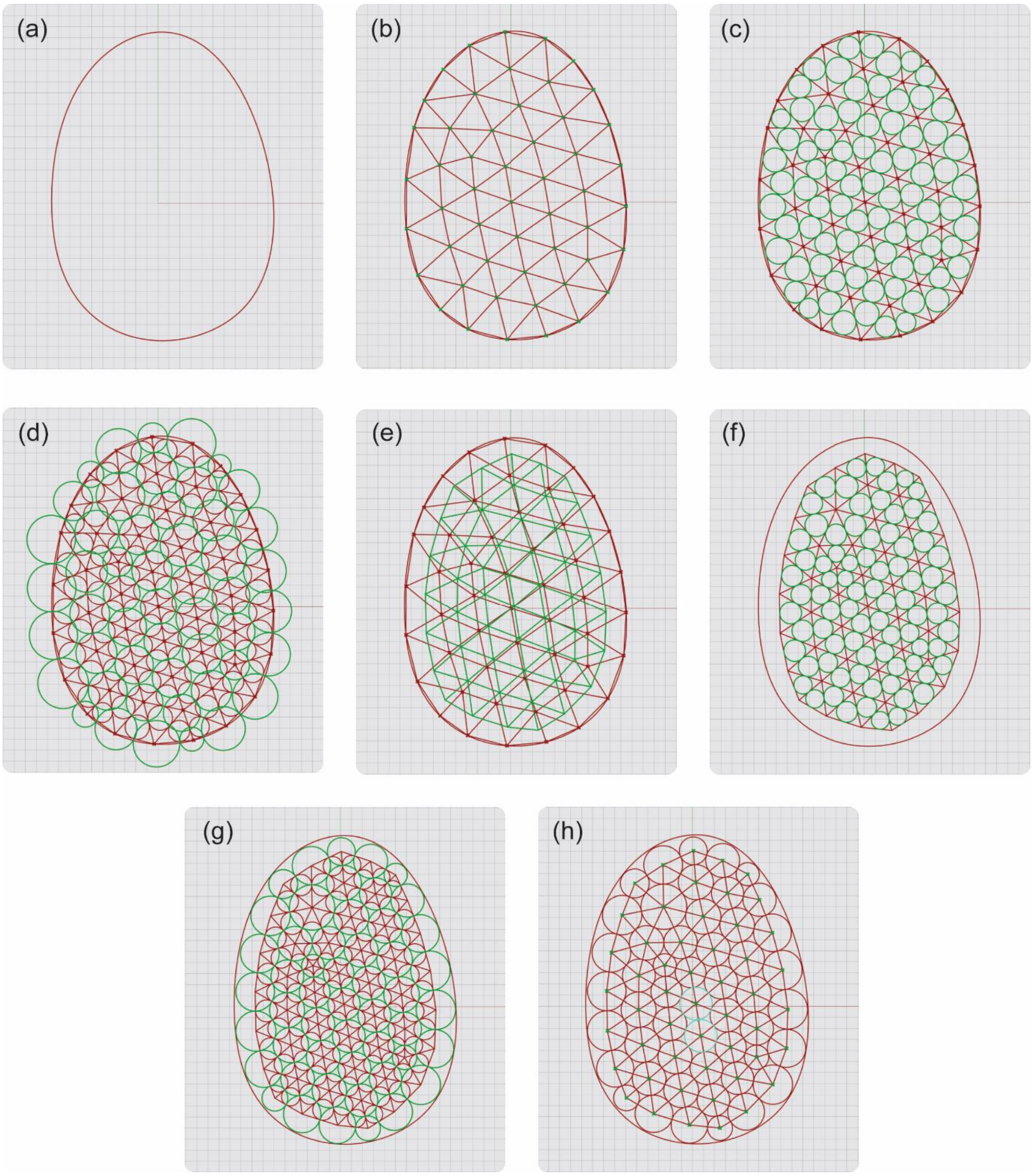

Figure S3.. Optimization process based on the triangle CP mesh for a plane with freeform boundary.

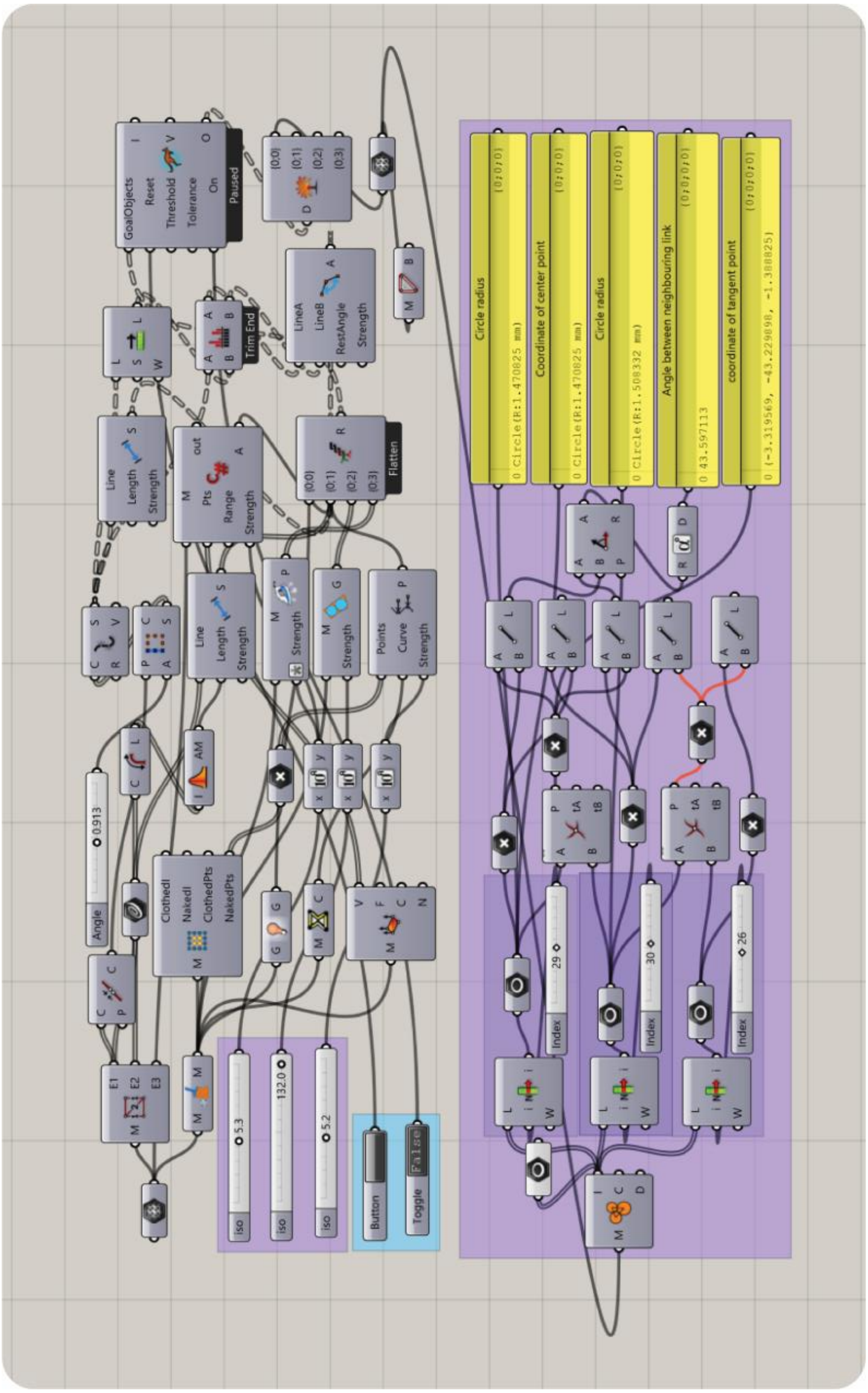

Figure S4. Function blocks diagram based on quadrilateral CP mesh in Grasshopper.

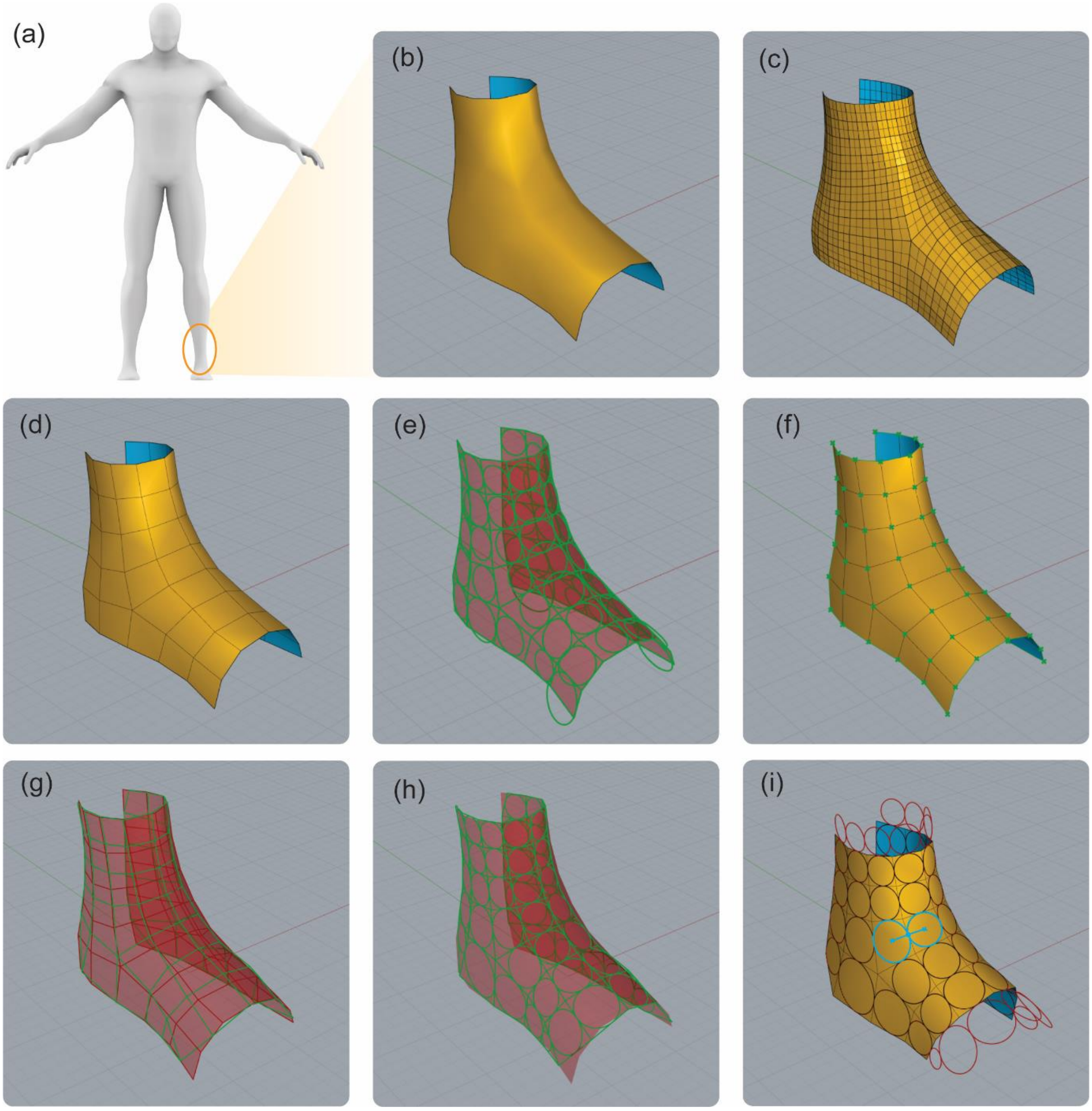

Figure S5. Optimization process based on the quadrilateral CP mesh for a freeform surface approximating the ankle cap configuration.

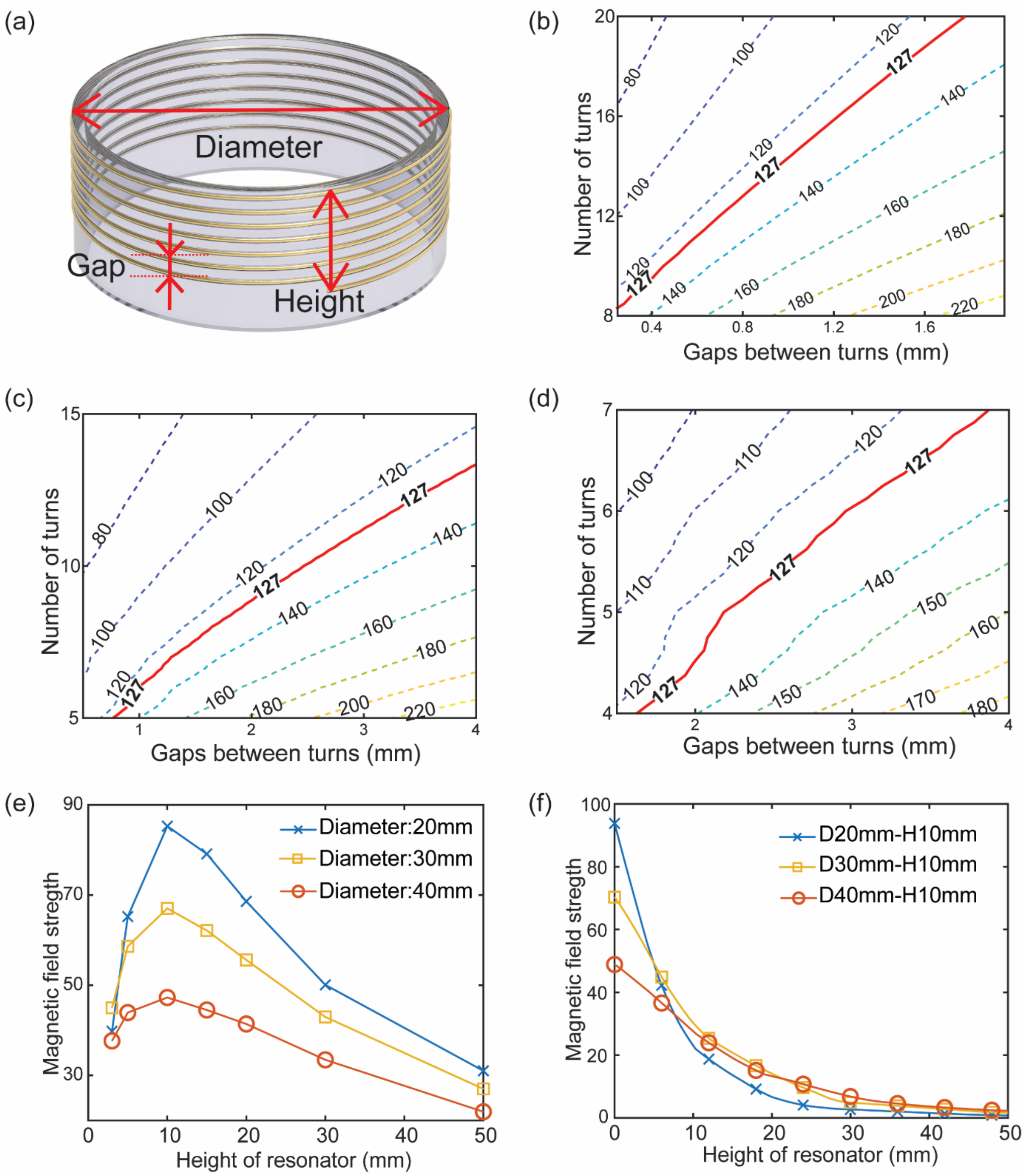

Figure S6. Optimization for HCRs. a) The configuration of an HCR. b-d) Resonance frequency of HCRs as a function of its configurations for HCRs with diameter of 20mm (b), 30mm (c) and 40mm (d). e) Resonance strength of HCRs with diameter of 20, 30, and 40mm by sweeping their heights. f). Magnetic field enhancement performance of HCRs with different configurations.

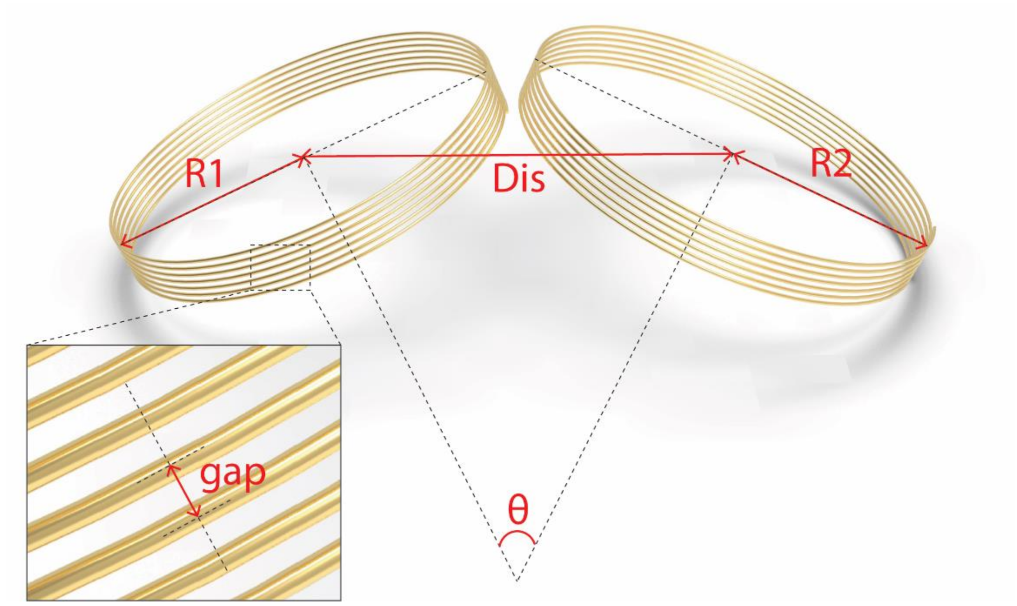

Figure S7. Geometric parameters employed in mathematical derivation for a pair of helical coils resonators.

(a)

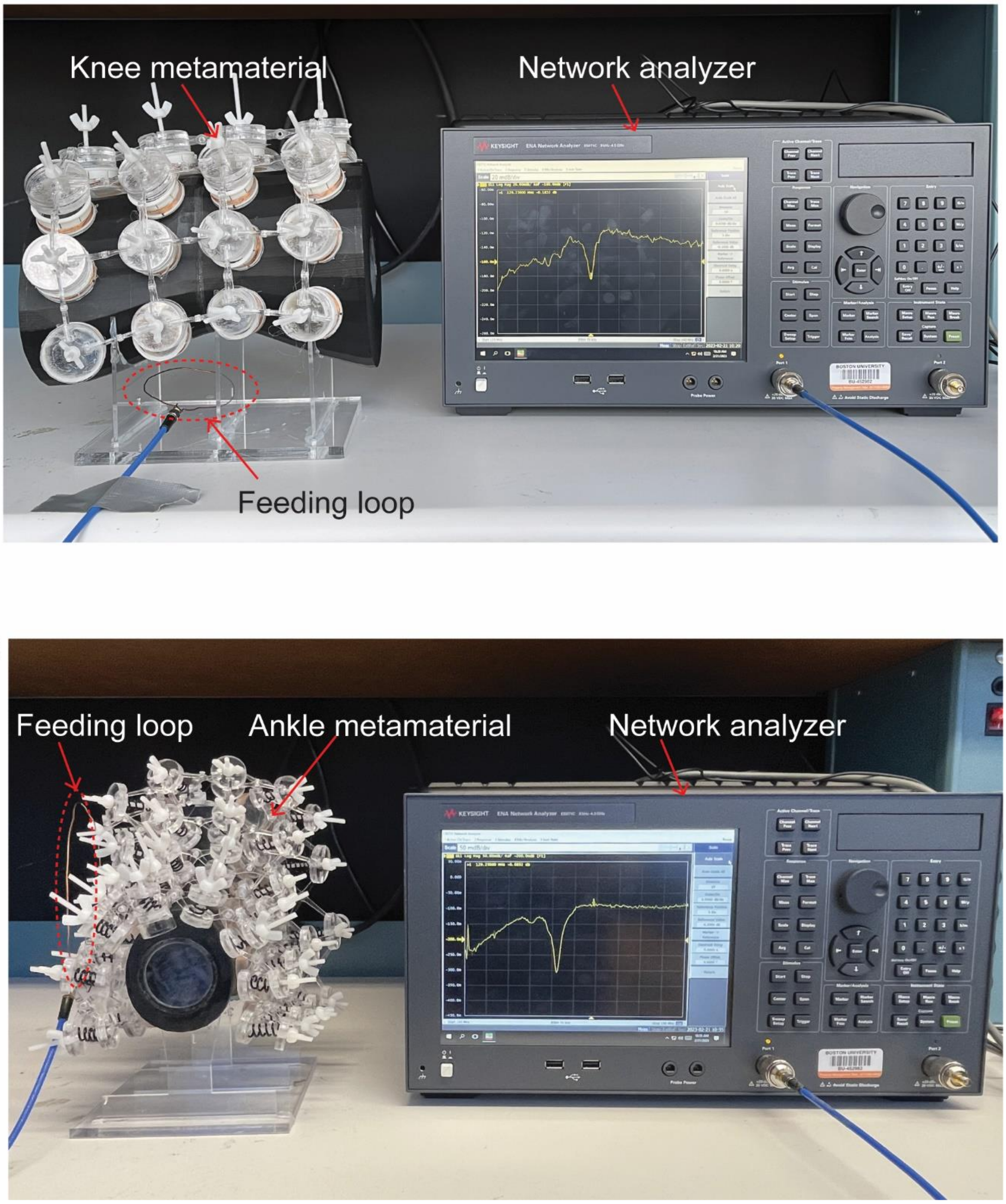

(b)

Figure S8. Experimental setup for the EM characterizations of knee metamaterial (a) and ankle metamaterial (b).

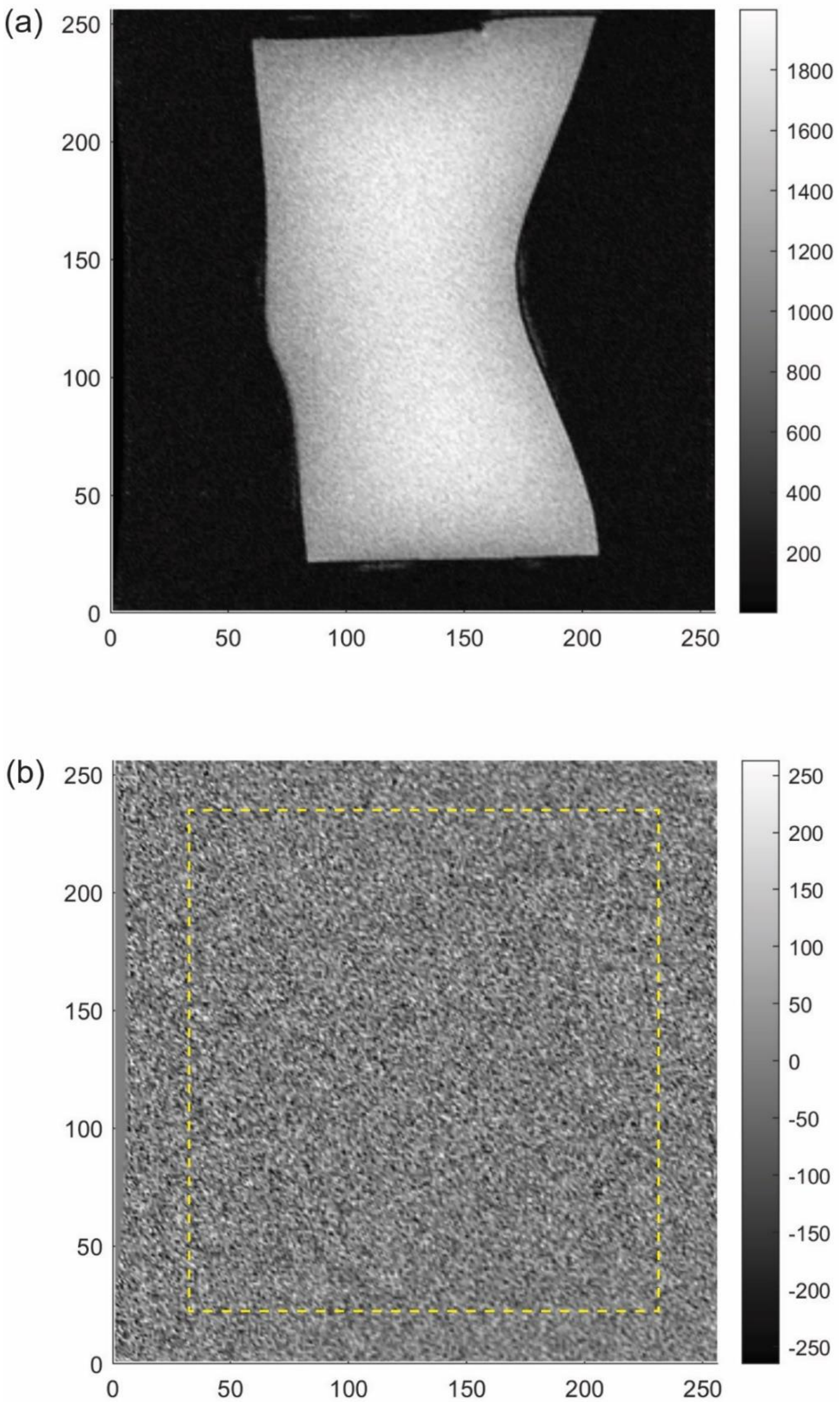

Figure S9. **Two-image method for SNR evaluation.** a) MRI image of the phantom, used to measure the signal. b) Image capture with the transmission amplifier off, the standard deviation of which (dotted black frame) was employed to derive image noise.

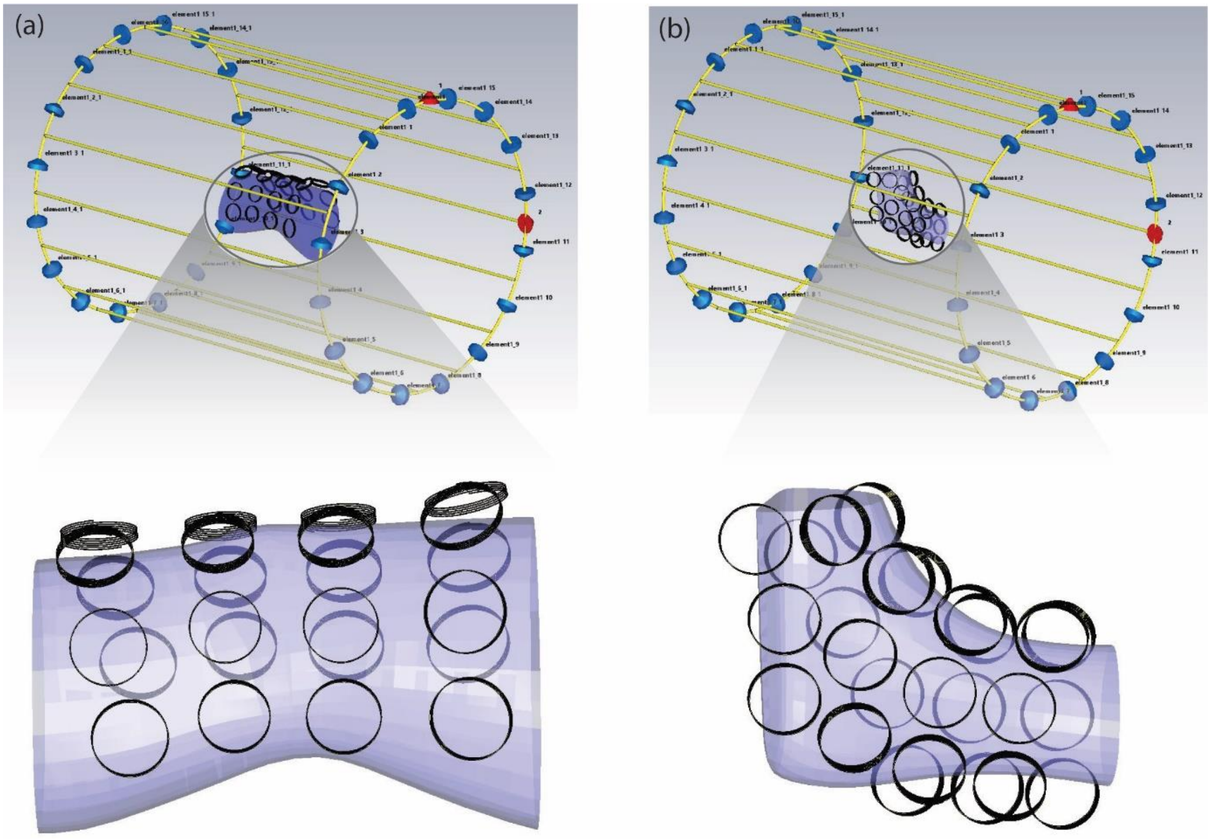

Figure S10. CST simulation model for knee metamaterial and ankle metamaterial.

Reference

[S1] K. Wu, , X. Zhao, , T. G. Bifano, S. W. Anderson, X. Zhang, *Adv. Mater.* **2022**, *34*, 2109032.

[S2] C. E. Hayes, L. Axel, *Med. Phys.* **1985**, *12*, 604.
